# Quality Improvement Initiatives in Reforming Patient Support Groups—Three-Year Outcomes

**DOI:** 10.3390/ijerph17197155

**Published:** 2020-09-30

**Authors:** Chieh-Liang Wu, Chia-Hua Liou, Shih-An Liu, Cheng-Hsu Chen, Wayne H-H Sheu, I-Ju Chou, Shang-Feng Tsai

**Affiliations:** 1 Department of Critical Care Medicine, Taichung Veterans General Hospital, Taichung 40705, Taiwan; cljeff.wu@gmail.com; 2 Department of Automatic Control Engineering, Feng Chia University, Taichung 40700, Taiwan; 3 Center for Quality Management, Taichung Veterans General Hospital, Taichung 40705, Taiwan; chliou@vghtc.gov.tw (C.-H.L.); andyliu7@gmail.com (S.-A.L.); yrc731@vghtc.gov.tw (I.-J.C); 4 School of Medicine, National Yang-Ming University, Taipei 11221, Taiwan; 5 Department of Otolaryngolog, Taichung Veterans General Hospital, Taichung 40705, Taiwan; 6 Division of Nephrology, Department of Internal Medicine, Taichung Veterans General Hospital, Taichung 40705, Taiwan; cschen920@yahoo.com; 7 Department of Life Science, Tunghai University, Taichung 40705, Taiwan; 8 Division of Endocrinology and Metabolism, Department of Medicine, Taichung Veterans General Hospital, Taichung 40705, Taiwan; whhsheu@vghtc.gov.tw; 9 Institute of Biomedical Sciences, National Chung Hsing University, Taichung 402, Taiwan; 10 School of Medicine, National Defense Medical Center, Taipei 114, Taiwan

**Keywords:** quality improvement (QI), patient support group (PSG), reform, healthcare, interdisciplinary

## Abstract

Background: Little has been done regarding the research on quality and quantity of patient support groups (PSGs) and how they can be improved. Here, we present three-year experiences of a quality improvement (QI) program of our PSGs. Methods: We launched earlier on a three-year project to improve our PSGs, including the number and quality of curricula. Data were collected on the number of PSGs, curricula, and participants. Results: In the first year, we organized relevant resources of our hospital and established a standard protocol for applying financial support and reporting the results. In the second year, we elected “the best patient” to promote sense of honor and better peer supports. In the third year, we surveyed through questionnaires participants’ health literacy to improve their feedback. Competitions and exhibitions of achievements were held each year to share results of every PSG. Finally, we had increased the volume of participation of patients and family over these three years (3968, 5401 (+35.5%) and 5963 (+50.3%)). Participation of staff also increased significantly (489 and 551 (+12.7%)). Furthermore, more interdisciplinary curricula were generated, with fewer doctors (38.2% to 29%), but greater numbers of the following: nurses (4.9% to 17.4%), nurse practitioners (0.4% to 14.5%), medical laboratory scientists (2.5% to 16.3%), social workers (4.7% to 41.7%), and teachers from outside (0% to 1.8%). Conclusion: In this first study on QI efforts on PSGs, we enlisted a core change team, drew a stakeholder map, and selected an improvement framework with good results.

## 1. Introduction

A patient’s outcome depends not only on medical treatment but also on supports from family members and peers. A patient support group (PSG) is a group of people with common experiences and concerns, and they provide emotional and moral supports for one another. PSG is proven to be beneficial for all kinds of diseases [1,2,3,4,5,6,7,8,9,10,11]. However, despite such positive value, engagement in PSGs is surprisingly low. A study reported that only a small proportion of patients (9.6% participated in face-to-face support groups and 4.4% participated in peer exchange online) engage in organized PSGs [12]. The phenomenon is likely due to reasons like limited resources (time and money) and extra-loading on medical personnel. After doing a thorough literature review, no article has yet been shared on how to initiate a quality improvement (QI) project in a hospital regarding how to improve the PSGs and curricula.

Stakeholders are those interested in a project [13]. Without doubt, physician engagement is particularly important for the success of PSGs [14]. For patient care, physician involvement is critical over healthcare outcomes and on improving healthcare delivery [14]. Therefore, most PSGs are composed of physicians but without evidence to show that physician involvement alone is the sufficient condition of good performance of PSGs. A successful performance of PSGs requires an interdisciplinary team and engagement of any other units of health care, including pharmacy, nursing, and administration. Collaborative efforts across multiple healthcare units can improve patient outcomes, healthcare processes, and levels of satisfaction [15]. In real life, most activities of PSGs are verbal communications or discussions led by physicians. In addition, activities from other team members are also helpful. Therefore, the engagement of other team members and adjustment of activities in PSGs should be encouraged to obtain better patient outcomes and satisfaction.

Quality improvement is a focused set of activities designed to monitor, analyze, and improve the quality of processes to improve performance [16]. QI programs are critical, because they improve patients’ outcome, efficiency of staff, and reduce waste due to failed processes. Not a single study has yet been reported on a hospital-based QI of PSGs, including organizations of PSGs and quality of curricula. In our institute, the Taichung Veterans General Hospital (TCVGH), we conducted a QI program over the last three years. This program included enlisting a core change team, drawing a stakeholder mapping and analyses, and using an improvement framework. Herein, we would like to share our experiences on this study.

## 2. Material and Methods

### 2.1. Methods

We presented the whole process of our QI collaborative efforts using data surveyed from PSGs. We also described and accessed projection outcomes. Since 2017, we started a QI initiative to reform PSGs in our institute.

### 2.2. Setting

The study was performed in the Taichung Veterans General hospital (TCVGH), where there are around 1500 beds and 5500 employees in this public medical center in central Taiwan. TCVGH provides safe, high-quality medical services with advanced facilities and training programs. The yearly numbers of patients were as follows: around 2 million for outpatients, 70 thousand for the emergent department, 47 thousand for operations, and 62 thousand for inpatients. It also provides patient-centered holistic care. Founded in July of 1986, the TCVGH Quality of Medical Care Committee was created for the evaluation and management of issues concerning healthcare quality. Most departments and divisions formed their own Healthcare Quality Improvement Circles. Since 2009, our hospital has implemented annual surveys regarding issues of patient safety and the culture of QI. However, the culture of QI for PSGs had never been done before.

### 2.3. Data Collection and Analysis

We collected data over three consecutive years (2017, 2018, and 2019), including numbers and names of PSGs, numbers of curricula, numbers of participants, satisfaction of teaching programs, and participation of healthcare providers.

### 2.4. Intervention

#### 2.4.1. Enlisting a Core Change Team

**Figure 1:**
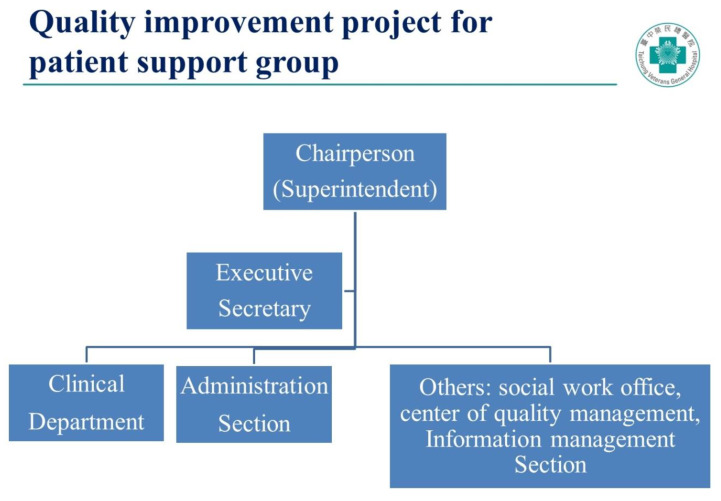
Core change team of quality improvement for patient support groups.

We used the Six Sigma model for the process improvement [17]. The first step of Six Sigma or any other QI models is to organize an executive core team [18]. Therefore, we enlisted a core change team for this QI project (Figure 1). Its chairperson was the hospital superintendent and the executive secretary, a physician. One staff from the Center for Quality Management was also recruited into this team. In addition, we had doctors from clinical departments, staff from the administration section, social work office, and the information management unit. This core team reviewed all results of PSGs every three months and made decisions about the priority of potential areas for improvement.

#### 2.4.2. Stakeholder Mapping

**Figure 2:**
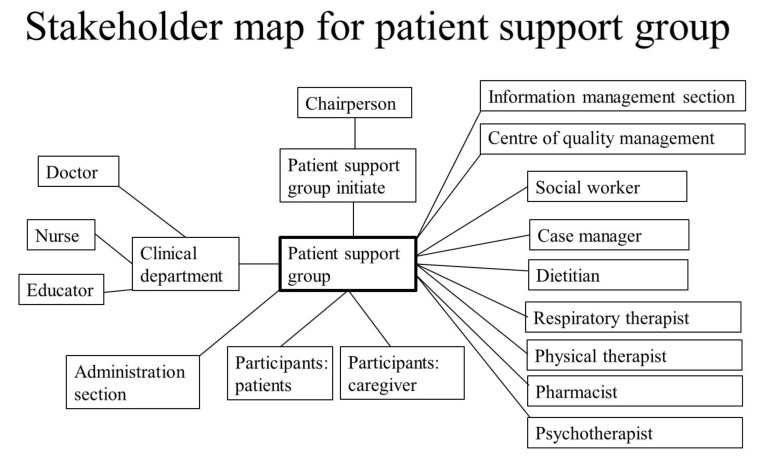
Stakeholder map for patient support group. The quality of a patient support group is shown at the center of the map, surrounded by various stakeholder groups.

We then used a stakeholder mapping to identify those who could affect the QI project (Figure 2). After that, we segregated stakeholders into different groups (like doctors, patients, caregivers, hospital leaders) and outlined their inter-group relationships. The quality element (PSG) was placed at the center of the map. First, the key points of PSGs were clinical departments (who recruited participants for PSGs) and participants (including patients and caregivers). Second, our executive core team for this QI (led by chairperson, superintendent) was also significantly associated with PSGs. Finally, some other stakeholders were associated with this QI, including the administration section, information management section, center of quality management, social worker, case manager, dietitian, respiratory therapist, physical therapist, pharmacist, and psychotherapists.

We analyzed various groups in terms of motivations, including barriers and facilitators. The team’s grand leader was the hospital superintendent, and leaders of individual teams (or individual PSGs) were the related department heads. The specialists of Quality Management and executive secondary were technical experts, who had full knowledge of all components of QI. Team members came from different areas of the healthcare system, but doctors and patients/caregivers played the most important role. About 35% of our physicians engaged in this QI for the purpose of obtaining better results, as in line with previous study [19].

#### 2.4.3. Selecting an Improvement Framework

After establishing the core team and stakeholder mapping, we used an improvement model to provide structures for diagnosing and treating this project. The three most popular models to consider were Six Sigma, Lean, and Model for Improvement [19]. We chose Six Sigma, which was developed by Motorola in the 1980s [17]. The choice was based on its best application to QI in which an immediate cause or solution could not be identified by the improvement team [17]. The improvement philosophy was a continuous improvement. This quality management system used five-phase processing (define, measure, analyze, improve, and control approach). We used the Plan-Do-Study-Act (PDSA) cycle as the tool for improvement [20].

## 3. Results

After establishing this QI program, we performed a survey on 45 PSGs from 25 departments (Table 1). In TCVGH, the yearly numbers of outpatients are around 2 million. However, only 45 PSGs and 60 activities were recorded as baseline status in 2017. The service of PSGs was low inappropriately compared to clinical service in TCVGH. In addition to the few numbers of PSGs and their activities, the quality was also unknown. PSGs for obesity, osteoporosis, and epilepsy of pediatrics did not have any activity in 2017. Most of their previous activities were not held regularly. More importantly, few data were recorded.

Discovering this shortage of data collection, we started to set up our three-year plan via PDSA (Table 2). First, our plan of this QI is to improve quality and quantity of PSGs. Therefore, a training program had been established. This two-way communication helped us to achieve the effect of health promotion. Second, a symposium with 46 participants was held with a 100% satisfaction rate. In that symposium, all leads of PSGs learned how the modeling group innovated ideas and led a cross-functional communication and collaboration. Third, all the above efforts had yielded many results and data. We then analyzed and studied them for qualification. Finally, through the above processes, we had achieved many milestones in this first year. By using that, we kept continuing to promote the activities of PSGs in various departments of this hospital, develop the organization of patients, and improve the health literacy of patients.

**Figure 3:**
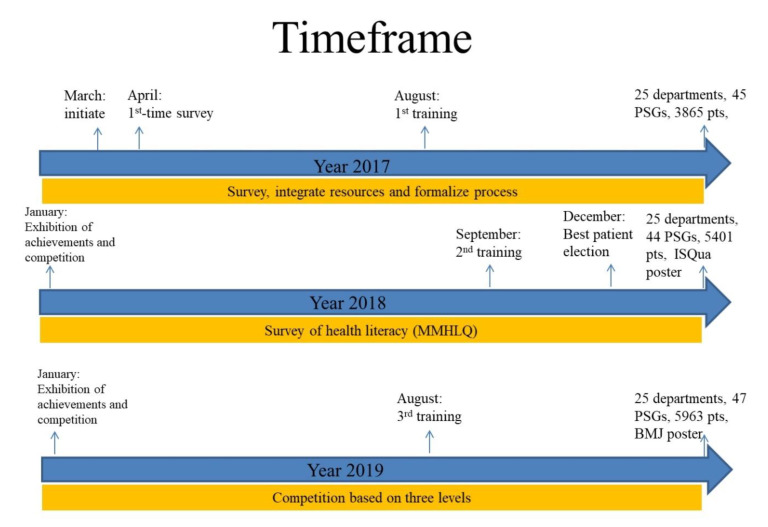
Timeframe of qualify improvement for patient support group. (The International Society for Quality in Health Care (ISQua); British Medical Journal (BMJ); patient support group (PSG)).

The entire time period is shown in Figure 3.

Here, in the first year, we initially surveyed the background status in our hospital: i.e., 45 PSGs with irregularly held activities. In this first year, we collected all data regarding PSGs in this hospital as baseline or standard. Then we informed all leaders of PSGs that we would have first-time training in August. All leaders were asked to join in the training meeting without any exception. We organized the resources of PSGs (places, money, and manpower) and informed all leaders of PSGs of our initiatives. We also standardized paper forms for application and records. The leaders were all informed that we need to gain cooperation from all PSGs. Items required for running PSGs were organized on a website (under our institute official website) and could be accessed by members of PSGs, including patients and caregivers. Before and after PSGs, announcements and take-home messages were also posted on the institute’s official Facebook page. All resources were organized with the aim of improving their efficiency in delivery.

In the second year, training was again done. Similarly, all leaders of PSGs were again asked to participate in this training meeting. We invited the top three best PSGs over the previous year to share their experiences on how to organize and handle PSGs and good ways to conduct PSG activities. The satisfaction rate was also 100%. In this second year, we again conducted a score-based election for “best patients”. The scores were based on the personality of optimistic, willing to share, supported by peer, good impact on peer and their PSG, sharing experiences with good fluency, and enthusiastic participation of PSG. The best patients received awards and later shared their honor with other patients. Similarly, a competition was also put up to include all PSGs which were invited to join an exhibition. During this second year, we intended to improve the quality of activities in PSGs. Therefore, we used the Mandarin Multidimensional Health Literacy Questionnaire (MMHLQ), which is a questionnaire for health literacy, to analyze participants’ needs for a PSG. At the end of this year, all participation had increased markedly from 2865 to 5401. We even gave a poster presentation (abstract No.: PO234) at ISQua’s (The International Society for Quality in Health Care) 35th international conference I, 2018, entitled “Promotion of patient support group to improve patients’ care”.

In the third year, we held the third training workshop and showed PSG participants the results we had collected from the MMHLQ. We discussed with every leader of a PSG as to how to increase patients’ health literacy in their PSGs. The top three PSG leaders also shared their experiences with members of this training workshop. We also held a competition for all PSGs. The participation also further increased from 5401 to 5963. At the end of the third year, we again gave a poster presentation at the International Forum on Quality and Safety in Healthcare 24^th^ conference ((jointly organised by the Institute for Healthcare Improvement (IHI) & BMJ)), entitled “The evaluation of health literacy among patient support groups in a medical center”. We then published a study, entitled “Health Literacy Varies According to Different Background Disease Natures and Characteristics of Participants for Patient Support Groups” [21]. We found out that the background status of participants varied according to different diseases. In addition, different disease natures and patient background statuses required different designs in PSGs.

As for the three yearly competitions of PSGs, scores were based on teamwork, unique characteristics, innovation of curricula, and the continuity of PSGs and skills of presentation. All PSGs were aware of the scoring system in advance, and they also learned earlier on what made a good PGS and ways to hold good activities. In the first two years, we chose the top four from all 45 PSGs to receive the monetary awards (330, 270, 200, and 10 USD, respectively). In the third year of competition, we revised the scoring rules. Additional scores were given if general physicians had been invited to participate in the PSG, and informed participants of the share-decision-making process. Initially, we divided PSGs into two levels. We awarded a total of 6 PSGs for their presentations. The first three PSGs were selected from those within the top 50% of all the ratings (with awards of 330, 270, and 200 USD, respectively). The last three were selected from the bottom 50% (with awards of 270, 200, and 130 USD, respectively).

During the studied three-year period of our QI program, we found marked improvements of PSGs (Table 3). Numbers of curricula had increased significantly (from 55, 105, to 118 per year). Besides, participation also increased significantly (from 3986, 5401, to 5963 per year). The participation of staff increased less significantly (from 489 to 551 per year), indicating close-to-very-efficient performance. Moreover, the involvement of multidisciplinary healthcare also improved significantly. The percentage of non-doctor participation of all staff increased significantly (no data in 2017, 61.8% in 2018, and 71% in 2019). Many more social workers were involved in PSGs (41.7% in 2019 vs. 4.7% in 2018). Psychotherapists (*n* = 2, 0.4%), coordinators (3, *n* = 0.5%), and outside teachers (*n* = 10, 1.8%) were all invited to join PSGs in 2019. Even though their participation percentages of all staff were not high, their involvement really improved the quality of PSGs.

## 4. Discussion

This is the first study to share experiences on how to perform a QI program to reform PSGs. We enlisted a core change team, drew stakeholder mapping, and selected an improvement framework. PDSA was used to organize all problems and to figure out the solutions. In the first year, based on background data of past years, we established our improvement policies, programs, and outcomes. Then, we modified them in the second and third years. We also created a learning organization to improve the quality of the PSG curriculum. In our opinion, the initial part of the program was of the greatest importance. The program needed assessment, analysis, planning, and implementation. The final step was creating quality assurance. We shared the detailed QI program to reform PSGs within a period of three years. Finally, we obtained better participation involving more disciplines.

The values of PSGs include patient education [8], sharing experience, peer support, decreasing anxiety [22], improving quality of life [23], improvement of medication adherence [11], and building-up of the doctor–patient relationship. However, there are some risks for PSGs in clinical practice. First, a huge daily workload and job stress both discourage staff from participating in PSGs. For example, according to a study in Taiwan from Chen et al. [24], the average workload of an attending physician is 65.6 h/week. The heavy workload included teaching, research, clinical work, and administrative work. The impact of a heavy workload on nursing staff also adversely affects patient outcome [25], which could discourage them from attending PSGs. Moreover, the allocation of limited health resources is always challenging [26], and the priority is not always placed on PSGs [27]. Therefore, even with the great value of PSGs, how to launch PSGs both effectively and efficiently becomes the major issue. However, the experience about how to reform PSGs is lacking in the current literature. A QI program involves systematic activities organized and implemented by an organization to monitor, assess, and improve its quality of healthcare [28]. Especially, better efficiency and less wasting in costs are the essence of QI. Thus, we organized this QI program to improve PSGs, which produced good outcomes. Typical examples for QI in healthcare are as follows: pharmacist-led medication therapy, optimizing sepsis care, and lowering length of stay by a systemic and data-driven approach. No reported study on a QI program for PSGs has been reported so far. This study is the first of its kind illustrating QI for PSGs.

We performed this QI program with the following basic concepts. First, we tried to establish a culture of QI for PSGs in our institute. Since 2009, our hospital has implemented annual surveys regarding issues of patient safety and the culture of QI. However, the culture of QI for PSGs had never been done before. We measured the baseline culture of QI for PSGs and assessed the change over time. The core team of this QI program was led by our superintendent, who supported and encouraged our QI efforts. Results from this core team were to be reviewed every three months, a procedure that reflected passions of the core practice team embracing quality. Second, we determined and prioritized potential areas for improvement. We identified problems and weak points of our PSGs, including number and quality of the curricula. In this part, we surveyed the baseline situations of PSGs from all departments and gained insights of the problems regarding optimal ways in launching the PSGs. Third, we collected and analyzed these data. This part was the core of QI, and our data facilitated our identification of potential areas for improvement. Then, we made decisions based on the data analyzed. Fourth, we circulated the data to all members of this QI program, including every stakeholder from all PSGs. All the information was transparent to all stakeholders. We then informed the entire team of our planning and implementation of the QI program in the form of a symposium. We communicated to all stakeholders regarding our project, goals, actions, and some preliminary results. Once we had good results, we informed all stakeholders to share the success. Fifth, in the second and third years, we showed every stakeholder our ongoing plan and results of evaluation. They also shared with others their experiences and suggestions. We were able to reevaluate our planning and intervention at every timeframe. Finally, we spread information on our successful results at the end of every year. For example, we shared what we had learned from this QI of SPGs at ISQua’s 35th international conference and International Forum on Quality and Safety in Healthcare 24th conference in the second and third year, respectively. The QI methodology we used was aimed to improve the PSGs.

## 5. Conclusions

The QI program, according to our experience, could be used to reform PSGs effectively. After a meticulous background check (identify internal and external resources), planning, and implementation, we found that PSGs had improved quality year after year. We concluded that the quality of PSGs can be reformed by QI.

## Figures and Tables

**Table 1:** List of patient support groups in the first year.

No.	Department	Patient Support Group (*n* = 45)	Numbers of Curricula or Activity of PSG (*n* = 60)	Time
1	Chest Medicine	Asthma	1	20 May
2	Respiratory Therapy	Chronic Obstructive Pulmonary Disease	1	18 November
3	Gastroenterology and Hepatology	Hepatitis	1	11 November
4	Endocrinology & Metabolism	Diabetes mellitus	3	27 June, 1 August, 16 September
5	Nephrology	Chronic kidney disease	2	22 July, 29 October
6		Transplantation	1	October 29
7		Dialysis	1	October 8
8		World Kidney Day	1	5 March
9		Autosomal Dominant Polycystic Kidney Disease	2	29 July, 18 November
10	Hematology & Oncology	Myelofibrosis	1	22 July
11	Rheumatology	Rheumatic disease	4	25 February, 11 March, 2 May, 1 July
12	Infection Disease	Human immunodeficiency virus	2	17 June, 19 August
13	General surgery	Breast cancer	2	25 March, 2 September
14		Hepatoma	1	19 November
15		Obesity	0	
16	Chest Surgery	Esophageal cancer	1	15 July
17	Urology	Prostate cancer	1	April
18	Colorectal Surgery	Colorectal cancer	1	1 November
19	Transplant Surgery	Transplantation	1	15 April
20	Orthopedics	Osteoporosis	0	
21	Obstetrics and Gynecology	Interstitial cystitis	1	August
22		Ovarian cancer	1	14 October
23	Pediatrics	Diabetes mellitus	2	26 August, 2 September
24		Hemophilia	2	26 August, 2 September
25		Epilepsy	0	
26		Heart	1	December
27		Williams Syndrome	1	5 August
28		Cancer	1	10 October
29		Fabry disease	1	June
30		Preterm	1	14 October
31	Psychiatry	Psychiatric	5	15 February, 19 April, 12 July, 23 August, 25 October
32	Ophthalmology	Retinitis Pigmentosa	1	23 September
33	Otolaryngology—Head and Neck Surgery	Laryngectomees	1	19 November
34	Family Medicine	Hospice	1	23 September
35	Stomatology	Oral cancer	2	13 May, 4 November
36	Neurology	Seizure	2	23 April, 26 November
37	Cardiovascular	Heart failure	1	25 March
38		Pulmonary hypertension	1	22 April
39		Atrial fibrillation	1	29 July
40		Post-cardiovascular surgery	1	6 May
41	Cancer center	Lung cancer	1	1 December
42		Gastric cancer	1	24 June
43	Geriatrics and Gerontology	The elderly	2	16 September, 24 September
44	Dermatology	Psoriasis	1	28 October
45	Physical Medicine and Rehabilitation	Rehabilitation	1	15 December

**Table 2:** Action plan.

Plan	There are a total of 45 PSGs in all of the 25 departments in the hospital. We had established a training program as well to teach each patient support group how to promote patient service, including creating new groups and activities. Such a program eventually helps patients get the correct concept of health care and disease treatment in the group interaction, as well as to promote two-way communication, achieving the effect of health promotion and adjusting to disease.
Do	On 28 August 2017, we held a symposium with 46 participants (professional support group staff) and achieved a 100.0% satisfactions rate. In the symposium, a good model from the patient support group network educated and shared experiences in funding the activities in peer learning. Also, the participants learned how the modeling group innovated ideas and led cross-functional communication and collaboration.
Study	All the above effort had yielded a total of 56 varieties of services, including educational materials, consultations, group therapy, team-building activities, and other resources, all of which were conducted by a total of 45 patient support groups within the 25 departments in 2017. A total of 3865 patients or family took part in these activities in 2017. A competition for the staff of each patient support group was held the following year (11 January 2018), and many factors were accessed for qualification. Team cooperation, uniqueness, novelty, and developmental potential were all given credits for evaluation.
Action	We had archived many milestones within just one year after the establishment of the training program. Currently, there was at least one patient support group dedicated to a specific department in the hospital. These departments held at least 2 events regarding patient support. In 2018, we will continue to promote the activities of patient support groups in various departments of the hospital, develop the organization of patients, and improve the Health Literacy of patients and the populace.
Do	On 28 August 2017, we held a symposium with 46 participants (professional support group staff) and achieved a 100.0% satisfactions rate. In the symposium, a good model from the patient support group network educated and shared experiences in funding the activities in peer learning. Also, the participants learned how the modeling group innovated ideas and led cross-functional communication and collaboration.

**Table 3:** The improvement of PSGs within 3 years.

All 25 Departments	2017	2018	2019
Patient support groups	45	44	47
Curricula	55	105	118
Participation of patients and family	3986	5401	5963
Participation of all staff	n/a	489 (100%)	551 (100%)
Doctor	n/a	187 (38.2%)	160 (29.0%)
Nurse	n/a	24 (4.9%)	96 (17.4%)
Nurse practitioner	n/a	2 (0.4%)	8 (14.5%)
Medical laboratory scientist	n/a	12 (2.5%)	9 (16.3%)
Social worker	n/a	23 (4.7%)	23 (41.7%)
Case manager	n/a	76 (15.5%)	77 (14.0%)
Respiratory therapist	n/a	4 (0.8%)	9 (16.3%)
Physical therapist	n/a	19 (3.9%)	23 (4.2%)
Pharmacist	n/a	3 (0.6%)	2 (0.4%)
Volunteer	n/a	118 (24.1%)	114 (20.7%)
Dietitian	n/a	20 (4.1%)	15 (2.7%)
Psychotherapist	n/a	0 (0%)	2 (0.4%)
Coordinator	n/a	0 (0%)	3 (0.5%)
Horticultural therapy	n/a	1 (0.2%)	0 (0%)
Teachers outside	n/o	0 (0%)	10 (1.8%)
